# Personal Trust and System Trust in the Sharing Economy: A Comparison of Community- and Platform-Based Models

**DOI:** 10.3389/fpsyg.2020.581299

**Published:** 2020-12-10

**Authors:** Sabine Gruber

**Affiliations:** Department of Economic Sociology, University of Trier, Trier, Germany

**Keywords:** system trust, personal trust, community-based production, Community Supported Agriculture (CSA), platform economy, Airbnb, risk management, degrowth

## Abstract

Currently, new business models created in the sharing economy differ considerably and they differ in the formation of trust as well. If and how trust can be created is shown by a comparison of two examples which diverge in their founding philosophy. The chosen example of community-based economy, Community Supported Agriculture (CSA), no longer trusts the capitalist system and therefore distances itself and creates its own environment including a new business model. It is implemented within rather small groups where trust is created by personal relations and face-to-face communication. On the contrary, the example of a platform economy, the accommodation-provider company Airbnb, shows trust in the system and pushes technological innovations through the use of platform applications. It promotes trust and confidence in the progress of technology. For the conceptual analysis, the distinction between personal trust and system trust defined by Niklas Luhmann is adopted. The analysis describes two different modes of trust formation and how they push distrust or improve trust. Grounded on these analyses, assumptions on the process of trust formation within varying models of the sharing economy are formulated as well as a hypothesis about possible developments is introduced for further research.

## Introduction

The question of how trust can be increased reaches a new stage as new platform technologies are introduced. Internet platforms offer a new range of possibilities to reach customers. While local marketplaces have previously been linked to face-to-face communication and analog interaction possibilities, nowadays, the communication between seller and buyer is no longer limited to such barriers. The opportunity to connect sellers and buyers from any place on the globe in real-time gave a boost to the industry, enabling new forms of exchange, such as the sharing economy. Platform applications make finding a match much easier and more efficient than before. An efficient way of matching sellers and buyers is an additional reason the popularity of sharing instead of owning currently experiences a revival (Botsman and Rogers, 2010; Rifkin, 2014).

In theory, platform economies represent a new level of system trust. Generally, we can differentiate between personal trust and system trust. In premodern societies, we knew our transaction partner personally and the number of business partners was manageable. This is impossible in modern societies. Therefore, personal trust has been replaced by modes of system trust that are impersonal and indirect. Markets are characterized by anonymous transactions. They are functional, although we have little information about our transaction partners and we can handle indirect communication quite well. However, internet communication opens up new opportunities for false information and distrust. The question of how to build trust in e-commerce and sharing economies is therefore of great interest.

This article discusses the question of building trust based on a contrast. It compares a business model based on system trust and a business model based on personal trust. Personal models of trust can be found in modern society, too. They are represented by the example of Community Supported Agriculture (CSA). In contrast, Airbnb is introduced as an example of system trust. While Airbnb would not exist without platform technology, CSA relies primarily on face-to-face communication. For the main distinguishing characteristics, see Table 1. CSA's philosophy may seem backward and may only reach a relatively small group of participants. Nevertheless, it can be considered successful and it relates to aspects of trust formation that are hardly replaceable. It is mainly the aspect of coherence between content and communication, and as it will be shown below CSA is very effective in minimizing the gap between its practice of risk sharing and its transparency in communication. Therefore, CSA teaches us about the essential principles of trust building which also matter at the system level. This paper aims to discover the decisive moments and generally applicable principles of trust formation.

**Table 1:** Organizational characteristics of the community-based and the platform-based example.

	**Community-based organization**	**Platform-based organization**
Communication	Face-to-face/interpersonal	Digital^1^/impersonal
Observation	Personal	Impersonal
Technology	Not constitutive	Constitutive
Members	Limited	Unlimited
Trust	In persons	In systems

1 *I use the term digital for a certain type of technology-enabled communication, for internet-based apps that are used on personal computers and smartphones, but not for phone calls or letters*.

The general question is: What can we learn about trust formation from a community-based business model and which indispensable conditions must be covered by both community-based and platform-based models. Before the models are presented in detail, Niklas Luhmann's theoretical framework on the social function of trust is presented and analytical categories are developed (section Personal Trust and System Trust). The examples are then illustrated using the theoretical categories (section Community Supported Agriculture as an Example of Personal Trust: CSA and section Airbnb as an Example for System Trust: Airbnb). The analysis of CSA is based on qualitative and quantitative data collected by the author (Gruber, 2020). Airbnb is covered by current research and literature. The analysis includes a description of the communication tools as well as an interpretation of the inherent trust-building strategy. In section Comparison the strategies are compared to not only establish the differences but furthermore the universal aspects of trust. In conclusion, hypotheses discussing the conditions under which personal or system trust become relevant are formulated (section Conclusion).

## Personal Trust and System Trust

As a theoretical framework Niklas Luhmann's early writing on trust is utilized. In his book “Trust and Power” (Luhmann, 2017 [1968]), his understanding of social action is characterized by using it as a category of action that leads toward a certain system. This differs from his later developed and more influencing theory, where his focus shifts from social action toward communication as a central category of his analyzes. His later theory on social systems can be read as a general theory of communication (Luhmann, 1984b). There, Luhmann introduces communication *as* social action which reduces complexity by selection. In his understanding this represents the main function of communication, which is independent of how the content is transferred (personal or impersonal). For the aim of this paper, his previous distinction of interpersonal and non-personal communication is applied.

The starting point of Luhmann's explanation is the function of trust—he derives the description of trust from its function. In order to demonstrate the function and to show that trust may be carried out in different ways, he puts the reasoning of trust in the context of the evolution of the social system. Throughout history we can observe that the social system became increasingly complex. Modern societies are characterized by a high degree of social differentiation and division of work, which makes personal knowledge of all members impossible. In large systems, there is no direct control over such processes, but there is an organizational need to reduce complexity, which is largely covered by trust as a central function: “All these ideas can be compressed into a single formula: in conditions of increasing social complexity, humankind can and must develop more effective ways of reducing complexity” (Luhmann, 2017 [1968], p. 8).

Niklas Luhmann aims to explain the rise in system trust, and that is why he differentiates between personal trust and system trust. Trust generally relies on the ability to explore the motivation for behavior. This opportunity of exploration is given in any situation where personal interaction takes place. As the opportunity for personal interactions is no longer available, other solutions have to be found, but this adjustment needs to be solved step by step. “The ‘principle of small steps’ replaces simpler forms of adaptation to the environment when the environment also operates in a contingent fashion or is too complex for adaptation at one stroke” (Luhmann, 2017 [1968], p. 45). Although Luhmann admits that there is still a desire for personal orientation, it has to be replaced by other mechanisms in complex systems. This mechanism is the use of information in a more selective manner. The point he makes for system trust is that the selection of relevant information is supported by exaggeration. This process takes place via media—therefore “(t)he function of a communication medium lies in transmitting reduced complexity” (Luhmann, 2017 [1968], p. 124).

Luhmann's most important finding seems to be the second point he makes. Reducing complexity through communication does not eliminate risk. Risk can only be reduced to a certain extent. To get an idea of how far risk can be reduced, another consideration is important which is the inevitable connection to truth. “Trust is only possible where truth is possible, where people can reach an agreement about any given entity which is binding upon a third party” (Luhmann, 2017 [1968], p. 57). Vice versa, if there is no truth, trust turns into distrust. Especially in the context of economic action, Luhmann sees some limitations of trust, or better a limitation in truth: in certain cases not truth but rather informal and unexpressed behavior is more functional than expressing the truth (Luhmann, 1964) to keep a profit-orientated, autopoietic system going (Luhmann, 1984a)^1^. Hence, we can understand the balance between truth and trust in economic actions as a fragile construction which can easily tip over.

Trust, truth and the reduction of complexity are very closely linked in Luhmann's theory. He also views it as connected to the general problem of uncertainty. According to Luhmann, trust helps us to solve the problem of uncertainty, not by elimination, but by compensation. It is compensated for by determining tolerable uncertainty. Via a communication process between two parties—e.g., a seller and a buyer—an agreement on the tolerable risk is made. In this manner, uncertainty becomes manageable. Luhmann is very clear about this: Not reality, but the image of reality is negotiable—and therefore uncertainty is tolerable.

The interrelation between trust, truth, and selective communication is a central insight that Luhmann gives us for this analysis of economic activities. For the comparison, we have to ask how tolerable uncertainty is established in small communities and in big systems, respectively. Following Luhmann, the examination of motivations in small communities lies in the responsibility of the individual, while in large systems the individual is relieved of personal examination and social control. Platform applications offer a substantial amount of relief for users. Hence, it is of high interest what kind of relief is offered and how successful it is. Some may lead to trust, some rather to distrust. In the following analysis, the advantages and disadvantages of these newly implemented communication tools will be discussed in detail using Airbnb as an example.

Examining Luhmann's approach, we can clarify the differences to other approaches in social science that deal with uncertainty and trust. They have limitations due to their focus on the market system and on impersonal communication as typical for markets. For example, Beckert (2002) discusses trust as an explanation for economic action because economic sociologists increasingly became aware that rational decision making is limited (Simon, 1972). Another important concept is that of reputation (Diekmann and Przepiorka, 2017). Reputation is applied as an explanation of trust in e-commerce (Dieckmann and Wyder, 2002), but originally it is based on perception. Its usage does not differentiate between reputation based on interpersonal or impersonal communication. By comparison, Luhmann's system theory is based on a historical derivation of trust formation processes and offers some differentiation to explain varying but parallel existing modes of trust.

Furthermore, the theory is chosen because it emphasizes the truth about how an enterprise deals with uncertainty. The coherence or incoherence between truth and the content of communication (interpersonal or impersonally transferred) gives us some crucial information about the likeliness of successful trust formation. How certain is it that an enterprise deals with uncertainty in a responsible way? What does its implementation of risk management actually look like and how does the enterprise address this? Are form (communication) and content (risk management) congruent? Respectively, can we plausibly check its correctness of what is done and what is communicated?

In order to cover all aspects of the trust-building process in the sense of Luhmann, the descriptions include the founding philosophy (motives of economic action), the business model (the implementation of the intended action), the communication strategy (personal/face-to-face or impersonal/digital communication), risk management (communication content), and the trust formation (coherency between selective communication and truthfulness).

## Community Supported Agriculture as an Example of Personal trust

### The Founding Philosophy of Community Supported Agriculture

Community Supported Agriculture represents a reaction to environmental problems which became apparent in the 1970s and which lead to the environmental movement in the 1980s. CSA and the environmental movement are strongly interrelated and they influence each other. The founders of the first CSA farms can be seen as key drivers of the ecological movement. The starting point of their criticism is the unsatisfying food supply of the agricultural industry. To begin with, industrial products are low in nutrients or even polluted and therefore unhealthy. Hence, the main issue of CSA is to provide people with healthy food. In Japan the pollution with pesticides is a very drastic example; it provoked the formation of Teikei—the Japanese equivalent to CSA established in the 1960s. The western model of CSA originates in Europe where pioneer farms were founded in Germany and Switzerland in the 1970s. In the mid-1980s Jan Vander Tuin and Trauger Groh brought this concept to North America and since then about 13,000 CSA farms have been founded in the USA and Canada.

All pioneers shared some particular worldview—they were critical of capitalism and open to spirituality. They placed their mission into the context of capitalism and industrialization:

“The many encouraging initiatives of Community Supported Agriculture (CSA) arise not in pastoral isolation, but rather amidst the vast and jangling context of global industrialization. The modern industrial processes of efficiency and mass scale have been brought to bear not just upon factories, but also upon a wide range of human activity, including our farms and our food. (…) At industrial farms and as it is handled by large corporations, food becomes a commodity, reduced to the physical properties (…). Through a focus on profitability, the soul of the land is excised and through petrochemicals, synthetic hormones, genetic manipulation, irradiation, and a host of other dubious materials and processes, the basic character of our food is altered” (Groh, 2000).

Consequently, in their opinion a “new thinking” (Groh, 2000) is needed which shall lead toward a non-exploitative production system.

This new model of agricultural production that they were fostering was implemented in a holistic, spiritual way of thinking. Although they did not follow one single religion, they were all familiar with the ideas of Ernst Friedrich Schumacher and Rudolf Steiner (McFadden, 2003). From the economist Schumacher (who was first influenced by Buddhism and later had a preference for Christian spirituality) they took the quintessence of his critical analysis, which became famous under the phrase “small is beautiful” (Schumacher, 2013 [1973]). They initiated their model by founding local farms and producing for locals rather than export their products. Today these ideas are discussed under the term of degrowth (Paech, 2012). Because Schumacher left little tangible instructions they were more inspired by Steiner. Rudolf Steiner created a whole new spiritual system, the anthroposophy, and left a holistically (more exactly syncretistic) philosophy as well as very detailed practical advice, not just for agriculture, but also for education, medicine, and many more fields.

Rudolf Steiner taught an agricultural methodology called biodynamics (Steiner, 1999 [1925]) which today is practiced under the name “Demeter.” It differs from bioorganic production mainly in its worldview; it interrelates its practices to cosmic ideas. Steiner also left behind writings on economic issues (Steiner, 2011). Inspired by his writings about the associative economy (Lamb, 2013) the pioneers created the consumer-producer association, which will be introduced in the next section. Essential to the founding philosophy is that social and economic goals, as well as environmental issues, should be balanced and harmonious.

Looking at today's motivations we can observe a shift toward less spiritual but more instrumental interests. Nonetheless, idealistic motives are still important to farmers and participants of CSA farms. While to the pioneer generation spiritual motives were most important, the new generation is mostly driven by political motives. This development corresponds with the environmental movement, which now occurs under the roof of transformation and refers to a broader socio-ecological context (Brand, 2016). Therefore, we can register a new ranking: the highest-ranked motive is “a socio-ecological transformation” (68.5%), however, the second important motive is still “following a holistic lifestyle” (57.9%). These motives are followed by an economic motive at the third place: “escaping the pressure of profit maximization” (57.0%) (Gruber, 2020, p. 53). The ranking is based on a survey conducted in 2018 in Germany, where the idea of CSA was reimported in 2008. From this time forward about 280 CSA farms were founded^2^. The renouncement of capitalism and the search for alternative modes of economic action is expressed by these idealistic motives, which are responsible for the engagement in CSA.

### The Producer-Consumer Association of Community Supported Agriculture

What can a business model look like that is not intended to promote economic growth but provides people with healthy and affordable food that is produced under fair social and ecological conditions? In general, there is no simple definition of CSA because the CSA community refuses to provide one recipe for all CSAs. CSA manuals introduce principles of CSA and leave the details of implementation open to each farm and its local preconditions (Wild, 2012, p. 9). Overall, the idea of small, diversified farms is promoted. Looking for a financing option to realize their vision, they came up with the idea of selling shares to a surrounding community. By signing annual contracts, they were able to collect money before the start of a season and finance the production work during this period. They called the concept “sharing the cost to share the harvest” (Henderson and van En, 2007, p. xiv). Starting with apples in 1985, the concept became the key model for CSA: the producer-consumer association.

The procedure of a CSA group usually follows typical steps with minor individual deviations: A general assembly takes place at the beginning of the year. At the annual reunion the production costs are presented. These costs are divided by the number of participating consumers; the result is the amount for one share. Then the consumers sign an annual contract that guarantees them a share of the harvest. Consumers often pay monthly and usually collect their share weekly. A share includes all grown fruits (or eggs, meat, etc.) depending on weather conditions and other circumstances that cannot be predicted. Throughout this process, products lose their character as a commodity, which is called decommodification^3^ and is discussed especially in the context of ecological transformation of the economy. Consumers fund the production work and not the products themselves (Wild, 2012, p. 9).

The main effect of this practice is that it ensures the financial basis of the farmers and at the same time ensures the supply of consumers with organic food. This creates a *win-win* situation for both sides. The concept undoubtedly requires a lot of engagement; anyhow, almost all CSA farms in Germany follow these principles—97.9% sign annual contracts and 97.9% produce organic food (Gruber, 2020, p. 62). In addition to financial support, consumers also provide active support to the farms—they help in the fields or administration. The extent to which consumers contribute actively varies a lot from farm to farm. Nevertheless, it is customary to pick up the share from farms or deposits (95.8%) (Gruber, 2020). All in all, we can summarize that CSA is a contract model between producers and consumers, in which consumers give an amount of money comparable to the prices of organic products in supermarkets, as well as some additional efforts for their active participation. The producers also face new tasks and have to reorganize their sales concept. They no longer sell their products to large companies, but to individuals or families. This requires new organizational and communicative skills.

### Face-to-Face Communication, Participation, and Transparency in Community Supported Agriculture

The willingness to participate in a CSA group, which is more time-consuming for the consumers and demands new management skills from the producers, is given because, on the one hand, the economic action is based on high-value orientation. On the other hand, communication plays a central role in demonstrating trustworthiness to consumers. Refusing the anonymous agro-industry, CSA establishes a model based on interpersonal relationships and a wide-ranging offer for face-to-face communication. Consumers want to know the producers of their vegetables personally and prove the origin of the products. Typical for CSA participants is their skepticism against certifications; instead, they prefer the personal contact to the farmers—or, as a farmer puts it in an interview: “My certificate is 200 families” (Gruber, 2020, p. 60, translation by the author).

The face-to-face contact strengthens trust in a certain way. Trust is built in situations in which we can examine the behavior of another person (Sztompka, 1999, p. 42). With personal trust the law of reunion governs the situation (Luhmann, 2017 [1968], p. 143) because we expect to see people again and therefore have to face their eyes: we cheat less but rather tell the truth. Although there are several varying communication options with CSAs, each CSA offers a certain space for face-to-face communication. Related to communication and participation opportunities, three types can be distinguished: the service-oriented, the participatory-oriented and the self-organized CSA—see Table 2.

**Table 2:** CSA types and spaces for face-to-face communication and participation (based on Gruber, 2020).

**Service-oriented CSA**	**Participatory-oriented CSA**	**Self-organized CSA**
Weekly pick-up days	Weekly pick-up days	Weekly pick-up days
Action days for fieldwork	Voluntary participation in fieldwork	Obligatory participation in fieldwork
	Voluntary participation in organizational work	Voluntary participation in organizational work
	Monthly plenary sessions open to consumers	Monthly plenary sessions open to consumers
Annual reunion with informative character	Annual reunion with participatory character (consumers participate in the decision-making process)	Annual reunion with participatory character (consumers participate in the decision-making process)
Events—e.g., celebration of Thanksgiving	Events—e.g., celebration of Thanksgiving	Events—e.g., celebration of Thanksgiving

The weekly face-to-face contact satisfies the desire for direct examination and personal trust-building. Furthermore, a significant factor is the transparency of the budget. CSAs present their annual calculations at the reunions, and some of them even publish them on their websites, which is an exceptional practice. Because there is complete transparency concerning the financial situation of a CSA, there is no room for misunderstanding. Another point that goes beyond trust formation is participation in decision-making. As far as it is practiced, decision-making strengthens identification with the enterprise. All in all, value orientation, interpersonal communication, transparency, and participation keep the producer-consumer associations close together.

### Bearing the Risk by the Community

The producer-consumer association is seen as a method for minimizing risks. For conventional farmers, risk is a big burden. They make all investments and do production work, but the yield depends on the final crop at the end of a season. Furthermore, prices depend on competition in the food market. Because price dumping on globalized food markets was ruinous for many farmers, the CSA model came up as a solution for them. The idea is to spread the risk throughout the community (Wild, 2012, p. 9). Covering the annual cost in advance by signing for annual shares gives greater security to the farmers. In this model the consumers take the risk with the growers, even if the crop fails. Pioneers talk about consumers as co-entrepreneurs. Although consumers of the new generation would not see themselves as co-entrepreneurs, they agree on bearing this risk. Sharing the harvest means sharing costs and risks, too.

In CSA the general business risk shifts from the farmer to the consumer. The reason for the acceptance lies, on the one hand, in the convictions of CSA participants, and on the other hand, is also motivated by practical reasons. They feel like CSA reduces complexity for them. What is included in a weekly share is determined by what is grown locally, hence there is no need to make a selection in the supermarket. This “service” is seen as a great relief from extensive demand. Several interviewees mentioned that they do not like to shop in supermarkets, and one interviewee expressed the advantage as followed: “You don't have a lot of trouble thinking about what you want to eat” (Gruber, 2020, p. 59, translation by the author). Participation in CSA represents a solution to the problem of reducing complexity in modern societies. Therefore, risk-sharing by the community is not only based on anthroposophical or social ideals, but also corresponds to more pragmatic interests.

### Trust Formation in Community Supported Agriculture

In this model of consumer-producer associations, trust formation is based on interpersonal communication. It is built on a community in which consumers and producers know each other personally. The confrontation of the risk-sharing strategy with the open communication strategy shows a maximum of accordance. Consumers are informed about the production costs (open budget) and the dependence of the share on the crop yield (annual reunions). They agree on this by signing up for an annual contract. The trustworthiness of the producers can be checked personally (at reunions, plenary sessions, pick-up days, and events on the farm). The congruence between communicated risk and practice leaves little space for doubt. Therefore, the high standard of personal trust-building fulfills the consumers' needs for control, which they miss in industrial production.

## Airbnb as an Example for System Trust

### The Founding Philosophy of Airbnb

Airbnb started as a survival strategy and became a global player setting trends for the sharing economy. In 2007, Brian Chesky and Joe Gebbia came up with the idea to sublet their apartment in San Francisco when they were hardly able to afford rent. They put three air mattresses in their living room and offered “Airbed and Breakfast.” In 2009 they were joined by Nathan Blecharczyk. The three turned their idea into a start-up company and founded Airbnb with a funding of 20,000 US-Dollar. As of 2020, Airbnb operates in 220 countries where 750 million accommodations are offered^4^ and possesses an operating budget of 450 million US-Dollar^5^.

The Airbnb success story became a role model for young start-ups and part of the identity of the Airbnb community. It is the story of “ordinary guys disrupting (the) industry” (Gallagher, 2017) and is addressed to ordinary people. When he was asked about his inspiration, Joe Gebbia said:

“For me, one of my personal inspirations was designers in the mid-twentieth century named Charles and Ray Eames. Their iconic furniture is in the Museum of Modern Art and it's still sold globally. One of the precedents of their work, one of the ethos of their work was to make the best design for the most people for the least price, and I feel like in some form or fashion, we've channeled a piece of the Eames thru Airbnb. By democratizing travel, by making it as accessible to as many people as we can by leveraging the power of the internet” (Tan, 2018).

To him, mass design in combination with quality and commercial distribution is beneficial. In his vision there is space for both idealism and commercial success.

The Airbnb founders present their idea as if there was no contradiction between self-realization and public interest; nevertheless, there is a difficult balance between economic and social goals. When Gebbia was asked: “Did you ever think the company would be in such hyper growth?” he answered: “I mean, in our wildest dreams” (Tan, 2018). Today they are under the top hundreds of richest Americans^6^. 2016 they joined the Giving Pledge—a philanthropic campaign launched by Bill Gates and Warren Buffett—and voluntarily committed to giving 50% of their earnings to charity projects. One cannot say that they are just driven by egoistic goals; nonetheless, there is a deviation between self-presentation and reality, i.e., they are creating a public image for themselves. Gebbia talks about democratizing travel by making it accessible. Accessibility is understood as using the internet to offer and find an accommodation (Büchner, 2016). Although the internet opens up many more opportunities, democratic participation is limited. At Airbnb the freedom to offer accommodation via the platform exists at the same time as the decision to book freely via Airbnb. This is the freedom of choice which is common on any marketplace and which should not be misinterpreted as democracy. On the peer-to-peer level, one can set the price and decide how much one is ready to spend, but on the company level, there is no democratic participation. It may be questioned whether the concentration of money and control among the owners of the platform is fair and to what extent this is represented in the public interest.

Airbnb users show a mix of financial and social interests. In tourism management, there is a particular research interest in the reasons for choosing an Airbnb accommodation over a hotel (Hennessey, 2014; Guttentag, 2015; Rimer, 2017; Tibulschi, 2017). Studies on the motives of Airbnb hosts and guests discuss the price argument, authenticity (Lamb, 2011; Bucher et al., 2017), collaboration and sustainability (Tussyadiah, 2015) as major motives. Daniel Guttentag gives us an impression of the ranking and user types. Based on a survey in Canada the three top motives are:

1. “For its comparatively low cost” (5.22 points from maximum 6 points)
2. “For the convenient location” (4.99 points), and
3. “For the access to household amenities” (4.70 points) (Guttentag, 2016, p. 108).

Referring to Guttentag we can distinguish between five user types:

1. Money savers,
2. Home seekers,
3. Collaborative consumers,
4. Pragmatic novelty seekers, and
5. Interactive novelty seekers (Guttentag, 2016, p. 126ff).

The most interesting result of his study is that although there is a diversity of motives and user types, low cost is the top motive (Guttentag et al., 2018, p. 13). Hence, Daniel Guttentag et al. resume: “This result also demonstrates that despite sharing economy rhetoric regarding ideals like sustainability and local consumption (e.g., Botsman and Rogers, 2010; Chase, 2015), it is the basic desire to spend less money that is often paramount” (Guttentag et al., 2018, p. 13). Although, if we take into consideration that the second important motivation is the type of location (i.e., its convenience) and that Airbnb users are open for new experiences and cultures, the decisive argument for their economic behavior still is cost.

### The Platform Model of Airbnb

The business model of Airbnb includes two innovations and both are new: what is rented and how it is rented. Renting out private rooms is described as peer-to-peer short-time renting (Guttentag et al., 2018). Because the peers are interlinked by a platform, a third party comes into action. Airbnb as a commercial company provides an online marketplace through which hosts and guests are enabled to find a match. Because hosts are not commercial sellers, we are facing a new construction between commercial and non-commercial actors. Nevertheless, money is charged for the accommodation as well as for the platform service, which causes a new situation that has not been provided with clear legal structures yet (Eichhorst and Spermann, 2015). While the legal handling is still unclear in detail, we can categorize Airbnb as a peer-to-peer for-profit company (Schor, 2014).

The online marketplace is implemented as a platform application. This platform (www.airbnb.com) covers tools for all steps of the customer journey from offering accommodation to finding a match, booking and payment. The website is assessed to be simple and straightforward (Guttentag et al., 2018, p. 2), which increases the usability of the platform. After an obligatory registration, hosts and guests generally find their way without additional advice. Using platform applications like this stimulates new business ideas. They follow a platform logic and can occupy new marketplaces (Kirchner and Beyer, 2016). Airbnb has been one of the first companies that used a platform in this way on a global scale; that is why Airbnb is often characterized as “disruptive innovation” (Guttentag et al., 2018, p. 2).

Platform economies come along with novel situations; however, their regulation is discussed after they have become operational. There are several regulatory issues such as competition, labor, social policy, taxation, insurances, etc. (Kirchner and Schüßler, 2020). Nonetheless, the contracting parties are clearly identified. One might think that hosts and guests sign one *single* contract with Airbnb, but it is a bit more complex as a separate corporation takes action for the financial procedure. Because of this, a four-person legal relationship exists between Airbnb's local subsidiary (e.g., USA, China, Japan, or the European Union), Airbnb Payments (with local subsidiaries), the host, and the guest (Treussl, 2018, p. 48). This way, Airbnb operates on a large scale. Using “the power of the internet” (Tan, 2018) Airbnb adopts the platform to local language needs and generates profit by collecting fees from hosts and guests.

### Digital Communication and Peer Reviews at Airbnb

The use of new technology challenges hosts, guests, and the platform provider. Trust has to be created under new communication conditions which are predetermined by the platform options. The platform provider has the role of the trustee—he wants hosts and guests to trust the mediation function of the platform. Hosts and guests are in the role of the trustors—they need to trust the platform as well as the other peer. Platforms confront us with the situation that they are poor in trust evidence (Jøsang, 2011) as they do not allow us to observe others personally; neither face-to-face communication nor telephone calls are intended. Platform communication is based on impersonal modes such as the presentation of self-descriptions online and the exchange of text messages. Therefore, we can assume a lack of interpersonal trust as a starting point (Tussyadiah and Pesonen, 2016; Sthapit and Björk, 2019). However, the question is which tools can be offered by a platform to increase trust.

In theory, three trust-building tools for e-commerce are mainly discussed: certificates, ratings, and reviews. While some experts have found certificates to be effective in proving trustworthiness because they are issued by an independent third party (Grimm et al., 2015), or they think ratings are functional because most transactions tend to be positive while negative ratings are the exception (Diekmann and Przepiorka, 2017, p. 689), others argue that additional reviews are required (Löwer, 2020). Therefore, the theoretical discourse is rather contradictory. In practice, star ratings and peer reviews are feasible. How well do they, however, meet the requirements of trust-building? To quote Luhmann: Can ratings and peer reviews convey a realistic picture necessary for system trust? The authenticity of ratings and reviews is questioned because they can easily be faked by invisible authors. Another argument is that peer reviews are more trustworthy than expert reviews (Löwer, 2020) because peers seem closer to the reality of the user than experts (or certification agencies). Nevertheless, they still represent an image, not reality.

However, peer reviews have become a favored communication tool at Airbnb. Nonetheless, there are some limiting aspects. They trigger a critical dynamic, as negative ratings and reviews have a greater influence on the decision of guests than positive ones (Riegler, 2018; Teubner and Glaser, 2019). Since users have to provide an Identification (e.g., a passport copy) the problem is not that it would be easy to pretend being a host or a gest, but the problem is that registered hosts as well as guests exaggerate. Some hosts present their home more comfortable than it is and some guest review their stay much worse than it was (as reported by effected users in the community area of Airbnb^7^). They drive the exaggeration too far so that the review does not refer to reality anymore. Being disconnected from an interpersonal experience it is much more likely that we put reality into a misleading light as it would be possible in a face-to-face communication. The result is that the host's reputation is damaged as well as the guests' trust. That is why peer reviews become suspicious although they are stated to be closer to reality than expert reviews. Another dynamic is that negative evaluation is avoid which leads to positive bias (Teubner and Glaser, 2019). Hence, complete compensation of reality via an internet conversation neither seems to be given nor possible.

For this reason, a study on the relevance of personal recommendations (word-of-mouth) for the purchase decision of Airbnb accommodations is of particular interest (Riegler, 2018). The confrontation of personal recommendations with online ratings has shown that personal recommendations are more important than online ratings. In individual cases, a personal recommendation even twisted the decision (Riegler, 2018, p. 63). Of course, this result should be checked for a general correlation. At the moment we can refer to another study on consumer behavior that shows that the willingness to share decreases with social distance (Schreiner, 2020). These results do not fully question the functionality of platforms, but they bring the need for interpersonal communication and personal orientation back to our minds.

It depends on how researchers put the question. If one just focusses on the platform economy as a subject and approaches the topic with a socio-technical theory, the result is that the technical standard is more effective in creating trust than personal recommendations are. Kong et al. have found, for example, that transaction security is about four times more effective in increasing trust than social referrals (Kong et al., 2020). It is also proven that the website quality can generally improve trust significantly (Wang et al., 2019). As opposed to these authors, I argue that we should not fail to see that those platform solutions are still faced with the desire for interpersonal observability (which is still satisfied in other contexts). The virtual and social reality is more interlinked than it may seem to socio-technical theorists. Especially before one first enters a platform, word-of-mouth might be more touching and influencing than an anonymous peer review.

This is why I suspect that even in systems like platform economies personal trust still matters. Consequently, tourism managers also argue for more interpersonal communication options at Airbnb: “(H)osts should engage in active communication with their guests … Positive online and offline communication may help develop trust between the host and the guest, as reciprocal interactions strengthen closeness and trust between two individuals” (Sthapit and Björk, 2019, p. 251).

Overall, impersonal communication shows some limitations, although it is functional. Trustworthiness can be improved by involving peers in online communication, and authenticity can be insured by providing pictures of hosts and guests (Broeder and Crijns, 2019). However, this does not fully compensate the basic need of face-to-face communication. Furthermore, communication with the providers remains open.

### Corporate Culture at Airbnb and Individual Risk Taking

When examining the discourse about building trust on platforms, it is worth noting that the focus is placed on trust between hosts and guests and the role of providers is not taken into account. What are the risks for hosts and guests, and what are the risks for Airbnb? Furthermore, who takes responsibility for these risks? Except for the payment, there are no legally binding rules. However, opening a private living quarter respectively staying at a private place is characterized by personal vulnerability (Sthapit and Björk, 2019), as there are neither binding rules on the hosts responsibilities nor on the rights and obligations of guests. Airbnb appeals to the users' honesty and hospitality, and introduces anti-discrimination guidelines, but there are little options to avoid violence, discrimination or even rape. There are no barriers before it is going to happen (O'Regan and Choe, 2017, p. 4). Airbnb can just block users after they showed misbehavior. Hence, there is no guaranteed standard what can be expected. Will the accommodation corresponds to the image of the website presentation? Will both parties show respect and behave in an appropriate manner?

In the first period of Airbnb, from 2009 to 2011, the business ran quite successfully and without major incidents, but in 2011, an act of vandalism shook Airbnb's image throughout the media. It sparked a response by Airbnb and a discussion about regulating Airbnb practices (Plott, 2014). Airbnb responded promptly and apologized to the affected host in public (Tate, 2011); furthermore, the company took responsibility and adopted its risk management. “In his blog post, Chesky announced a 24 h hotline^8^, a doubling of the support staff, and a ‘$50,000 Airbnb Guarantee’, which covers ‘loss or damage due to vandalism or theft caused by an Airbnb guest’ (Tate, 2011). The host guarantee became part of the corporate culture^9^. Although there are exceptions to what is covered, hosts can request support if they become victims of vandalism. The internal regulation clearly determines what is covered by the guarantee and what is not (property damage is covered, the loss of cash is not). Thereby, the level of security has been significantly improved. In addition, Airbnb's “belonging initiative” has been launched^10^. It promotes Airbnb's mission and encourages the users' good behavior and hospitality. The hosts are invited to share their experiences and best practices in meetings and on the blog. In this way, Airbnb is aware of its responsibility and sets practical consequences. The corporate culture developed by Airbnb can be seen as a reason for fewer conflicts than what other platforms are faced with such as Uber, which shows less respect for complaints and legal authorities than Airbnb (Sundararajan, 2014; Gruber, 2019).

However, surveys still show that hosts are not satisfied with their financial and material security (Malazizi et al., 2018). The reason for the general dissatisfaction lies in a more complex situation. Hosts are involved in questions about income, social insurance and taxation—questions that are left up to local authorities. Airbnb does not declare responsibility for these questions, and therefore hosts feel left alone. Although there is a consciously promoted rhetoric of corporate identity and moral standards, legally, peers are just part of the payment regulation. Therefore, a lot of risks are individualized in this model. Tourism managers ask for better reachability and clear definitions of a host's responsibilities (Sthapit and Björk, 2019), but satisfactory risk management would have to go beyond this. It would have to solve the gray area in between the triangle of market players (Airbnb company), legal authorities and civil society (hosts and guests). This is no easy task at the moment, but it is necessary if the content of risk solving and rhetoric should become more uniform.

### Trust Formation at Airbnb

The trust formation process at Airbnb takes place in a complex system between unknown peers via digital communication. Elaborated tools are offered in order to make internet communication as satisfactory as possible. The trustworthiness of the transaction partner can be improved by peer reviews. The indirect process appears to be quite functional, but when peers have the option of a personal recommendation, they give it priority. As Airbnb is based on internet technology, trust in the process of matching hosts with guests must be demonstrated through impersonal communication and images. The difficulty lies in verifying the content and therefore the truth of the images. Although the function of images is to reduce complexity in large systems, a problem appears as soon as there is a difference between selected communication and risk management, which seems to be the case in part at Airbnb. It has been announced that Airbnb cares about the security of its users, and some risk management tools are firmly set. Financial compensation is offered for acts of violence, but most tools appeal to a hosting culture. Therefore, security largely depends on individual behavior and not on legally binding rules. It is rather difficult for the individual to check the trustworthiness of their transaction partner—of the other peer and the platform. One reason is that there is no possibility of interpersonal observation or communication, another is that the contractual situation is quite complex and not transparent. There is a gap between the rhetoric of belonging and legal integration, which can hardly be checked by users. This incoherence leads to an irritating feeling of dissatisfaction or even to distrust, but for the majority of the users the residual risk appears tolerable.

## Comparison

Community-based and platform-based models show very different conditions for trust formation. They are built on different motivations and choose different realization contexts. CSA farms are founded as a reaction to distrust in industrial agriculture. They establish a counter-model to capitalism that follows socio-ecological goals by local farming. Airbnb does not go into opposition to capitalism but arises from mainstream thinking. The founders adopt the idea of mass design for their business concept, making accommodations accessible for most people via the internet. According to their vision they implement different business models. The key model of CSA is the consumer-producer association, a contract model that ensures the farmers' earnings and the consumers' supply with organic food. Complexity is reduced by embedding the production and distribution in small groups. In this way, a *win-win* situation is created with no need for further profit-making. As opposed to CSA, Airbnb relies on another business strategy. A maximum amount of users can be reached by implementing a platform application. The internet service offers a suitable matching tool for hosts and guests for which user fees are charged. The business model turned out profitable and practicable on a global scale.

In principle, both models are functional but they differ in complexity, scale and susceptibility to distrust. Reducing complexity by embedding economic action in small groups leads toward more trust but little scale, while operating on a large scale lacks satisfying communication and confidence. The reason for this can be found by the examination of the coherency between risk management and selected communication. The consumers carry risk in both models; in CSA farms the crop is uncertain and at Airbnb the quality of the accommodation, as well as the guest's and host's behavior, cannot be guaranteed. The decisive difference is transparency, that is, how clearly the truth is expressed about real risks and possibilities of risk compensations. In CSA there is full transparency regarding the financing and production process. The reputation of the responsible farmers can be examined via personal perception. Personal involvement is time-consuming but results in high confidence in the production process and the conscious acceptance of bearing the risk in case of a crop failure. At Airbnb the complexity of the many anonymous users cannot be reduced in this way. Under these circumstances, selected and impersonal communication is focused on the image of belonging and hospitality. The irritating aspect is that these images hide some limitations of risk protection. Some risks are compensated by Airbnb, some would be the responsibility of local authorities, and most risks are left to the individuals and their sense of responsibility. The difficulty is that personal behavior cannot be examined or compensated for by peer reviews, and the incomplete information carried by the images leaves the users with an unsatisfying feeling. That is why Airbnb can manage the online matching service on a big scale, but compared to CSA the model generates less trustworthiness and satisfaction.

## Conclusion

The article examined trust formation under the premise of congruent communication of risk. As all economic action is related to uncertainty, risk management has to take place. In practice, full risk control is never given, therefore trust comes into action as a compensation strategy for uncertainty. In this context, trust can be analyzed as a coping strategy reducing complexity. As complexity increases in modern societies, the distinction between personal trust in small communities and system trust in large systems was made. By making this differentiation we learn that the community-based example shows clarity in risk-sharing as well as transparency in communication, while the system-based example does not make individual risk explicit and cannot grant for true content or personal observation. Therefore, trust, coherency, and cohesion are higher in the community model. The system model is functional too, but it stresses the limits of trust formation, because it is relatively poor in trust evidence. Nevertheless, the example of Airbnb also proves its trustworthiness to its users. It represents an example of system trust and trust in technology and mass communication. In contrast, CSA users withdraw their trust in the agricultural industry and establish a countermodel. Summing up the two diverging processes of installing an innovative business model, the following assumption can be formulated: Personal trust and face-to-face communication still matter at the system level, because the need for personal orientation cannot be fully compensated by digital tools. The compensation of personal trust via impersonal communications is possible but only as long as images do not differ greatly from true content. Further research can thus examine the following hypothesis: If the gap between images and truth becomes too big and therefore distrust rises, one possible reaction is the return from the system level to a group level and consequently a move toward personal trust formation.

## Author Contributions

The author confirms being the sole contributor of this work and has approved it for publication.

## Conflict of Interest

The author declares that the research was conducted in the absence of any commercial or financial relationships that could be construed as a potential conflict of interest.

## Footnotes

^1^For example, a company's press officer is not expected to speak the truth unconditionally (e.g., about environmental protection measures), but rather present the company's activities in the best possible light.

^2^For the actual number of CSA in Germany, see: https://www.solidarische-landwirtschaft.org/solawis-finden/auflistung/solawis/.

^3^For decommodification in general, see Essays (2018), for decommodification and ecology, see Gerber and Gerber (2017), and for decommodification at CSA, see Boddenberg et al. (2017).

^4^https://news.airbnb.com/en-us/fast-facts/

^5^https://en.wikipedia.org/wiki/Airbnb

^6^Brain Chesky is listed #437, https://www.forbes.com/profile/brian-chesky/?list=billionaires#690f742444d3, 2020-06-13

^7^https://community.withairbnb.com/t5/Hosting/Let-s-talk-about-fake-reviews/td-p/1031509

^8^The hotline is implemented as an e-mail service.

^9^https://blog.atairbnb.com/host-guarantee/

^10^https://blog.atairbnb.com/belong-anywhere/
